# From Passive Surveillance to Response: Suriname's Efforts to Implement Maternal Death Surveillance and Response

**DOI:** 10.9745/GHSP-D-20-00594

**Published:** 2021-06-30

**Authors:** Lachmi R. Kodan, Kim J. C. Verschueren, Geertje Boerstra, Inder Gajadien, Robert S. Mohamed, Lily D. Olmtak, Satish R. Mohan, Kitty W. M. Bloemenkamp

**Affiliations:** a Academic Hospital Paramaribo, Paramaribo, Suriname.; b Department of Obstetrics, Division Women and Baby, Birth Centre Wilhelmina's Children Hospital, University Medical Centre Utrecht, Utrecht University, Utrecht, The Netherlands.; c Pan American Health Organization, Paramaribo, Suriname.; d Bureau of Public Health Suriname, Paramaribo, Suriname.; e Ministry of Health Suriname, Paramaribo, Suriname.; f Diakonessen Hospital, Paramaribo, Suriname.; g 's Lands Hospital, Paramaribo, Suriname.

## Abstract

Implementation of maternal death surveillance and response (MDSR) is crucial to reduce maternal deaths. In Suriname, MDSR was not implemented until 2015. We describe the process of MDSR implementation in Suriname and share the “lessons learned,” as experienced by the health care providers, national maternal death review committee members, and public health experts. Before 2015, maternal deaths were identified using death certificates and by active surveillance in the hospitals. Based on the recommendations from a 2010–2014 Reproductive Age Mortality Survey in Suriname, a maternal death review committee has improved the identification of maternal deaths and has audited every death since 2015. Although this review committee initiated several actions to implement MDSR together with health care providers, the involvement of the Ministry of Health (MOH) was crucial. Therefore, the Maternal Health Steering Committee was recently installed as a direct working arm of MOH to guide MDSR implementation. One of the main barriers to implementing MDSR in Suriname has been the lack of action following recommendations. Delineating roles and responsibilities for action, establishing accountability mechanisms, and influencing stakeholders in a position to act are critical to ensure a response to the recommendations. To implement MDSR, the 5 Cs—commitment, “no blame, no shame” culture, coordination, collaboration, and communication—are crucial.

## Abstract (precis)

To implement Maternal Death Surveillance and Response successfully in Suriname, recommendations to reduce maternal death should be acted upon. Delineating the roles and responsibilities for action, establishing accountability mechanisms, and influencing stakeholders in a position to act are critical to ensure a response to recommendations to avert maternal mortality.

## Key Messages

- Crucial elements for a successful maternal death surveillance and response (MDSR) implementation are the 5 Cs: commitment, “no blame, no shame” culture, coordination, collaboration, and communication.
- Essential steps to achieve sustainable MDSR in Suriname are: (1) improving the identification and notification of maternal deaths and (2) introducing and continuing systematic facility-based and national maternal death reviews.
- Lack of action following the recommendations to reduce preventable maternal death is the biggest obstacle for MDSR implementation in Suriname.

## BACKGROUND

The reduction of maternal deaths was the focus of Millennium Development Goal 5 in 2000, and it remained a priority in the Sustainable Development Goals established in 2015.^1^^–^^3^ In addition to counting maternal deaths, it is essential to identify underlying causes and contributing factors to gain more insight into the gaps in care to prevent avoidable deaths.^4^ A maternal death review is a medical audit with an in-depth qualitative investigation of the causes and circumstances of death.^5^

The Maternal Death Surveillance and Response (MDSR) cycle is a continuous action cycle that provides information on maternal mortality surveillance and audit and on the actions needed to improve care and avert avoidable maternal deaths.^5^^,^^6^ The World Health Organization (WHO) introduced the MDSR approach in 2012 to establish accurate data collection and to translate “lessons learned” to action plans and national policies, followed by monitoring to capture the effects.^7^ In Latin America and the Caribbean, MDSR was implemented in 2015 in 6 countries: Brazil, El Salvador, Colombia, Jamaica, Mexico, and Peru, which now serve as examples for other countries.^8^

**FIGURE 1:**
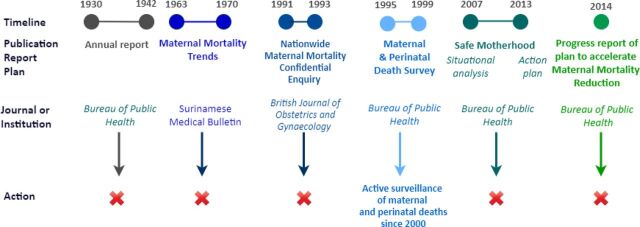
Timeline of Local Plans to Reduce Maternal Mortality in Suriname up to 2015

Although the maternal mortality ratio declined with time from 226 per 100,000 live births in 1991–1993 to 154 per 100,000 live births in 2010, Suriname was designated by the Pan American Health Organization (PAHO) in 2010 as one of the 10 priority countries in Latin America and the Caribbean for reduction of maternal mortality.^9^^–^^11^ Several intentions existed to improve surveillance and classification in Suriname for years, but integrated reviews of maternal deaths were not performed until the installation of a national maternal mortality review committee (the committee Maternal Mortality Suriname [MaMS]) in 2015 (Figure 1). We describe MDSR implementation in Suriname and its facilitators and barriers. We share the lessons learned, as experienced by the health care providers, committee MaMS members, and public health experts involved in MDSR implementation. This MDSR process is described for 3 time periods: (1) pre-2015, a history of MDSR and safe motherhood initiatives before the installation of committee MaMS, for which we conducted a review of key documents; (2) 2015–2019, during the MDSR implementation process, for which we describe the experiences of involved stakeholders (health care providers, committee MaMS members, and public health experts); and (3) 2020 and beyond, the way forward to fulfill the MDSR cycle, for which we describe the strategies of the Ministry of Health (MOH) based on the recommendations and experiences of the previously mentioned stakeholders.

## MATERNAL DEATH SURVEILLANCE AND SAFE MOTHERHOOD INITIATIVES BEFORE 2015 IN SURINAME

Suriname is a middle-income country in South America with 583,200 inhabitants.^12^ The country has an average of 10,000 births in a year, with 86% occurring in the 5 major hospitals, 6% in primary care, 4% at home, and 4% at an unknown location.^13^ MOH coordinates the health care systems in Suriname. The Bureau of Public Health (BOG, Dutch acronym) is responsible for public health programs and manages the surveillance and analysis of health data. Although every hospital collects data on maternal health key indicators, no comprehensive national health information system exists.^14^

Figure 1 presents a historical overview of the initiatives conducted to improve maternal health care in Suriname up to 2015. To obtain information on the history of MDSR, we collected and reviewed all documents on maternal health in Suriname available online and those provided by MOH, BOG, and the library of the Anton de Kom University of Suriname. Most of these documents were unpublished. The registration of deaths in Suriname began in the 19th century, but only for inhabitants who were not enslaved.^15^ An official civil registration system has been in place in Suriname since 1917, and vital events, including births and deaths of all inhabitants, are registered.^16^ The Central Bureau of Civil Affairs is responsible for civil registration. Death notification and registration are done using a death certificate, which is the responsibility of BOG.^17^ Underreporting of maternal deaths often resulted from death certificates not being completed or being completed long after an individual's burial.^10^^,^^18^^,^^19^

The first reports on maternal mortality did not provide information on the procedure for identifying maternal deaths.^20^ A confidential enquiry into maternal deaths, conducted by Mungra et al.^11^^,^^18^^,^^19^ in 1991–1993, highlighted severe underreporting (63%) and made the call to MOH to improve surveillance by undertaking active surveillance, implementing a Reproductive Age Mortality Surveys (RAMoS), and performing maternal death reviews. In 2000, BOG initiated active maternal death surveillance through a monthly enquiry in all obstetric units in hospitals.^21^ The cause of death was assigned by the attending physician. Since neither a multidisciplinary review nor a maternal death classification existed, every death in pregnancy (including deaths due to coincidental and accidental causes) was considered a maternal death.^10^ However, deaths in the antenatal or postpartum period of women admitted to nonobstetric wards were not captured.^10^

To reduce maternal and perinatal mortality, the MOH performed an analysis in 2007, followed by different action plans and reports, each presenting similar recommendations, but the plans were not implemented.^22^^–^^24^ Implementation was less successful than intended due to a lack of human resources, poor communication, and scarce coordination.^23^^,^^25^ Additionally, these plans and reports were little known among health care providers and other important stakeholders needed for the response.^23^^,^^25^ Surveillance had barely improved since 2000 and maternal death audits were not conducted until mid-2015.^10^^,^^21^

In 2015, a RAMoS was performed by health care providers to retrospectively identify and audit all maternal deaths between 2010 and 2014.^10^ An array of methods was used to identify pregnancy-related deaths, as described in previous publications.^10^^,^^11^^,^^19^ Various medical experts determined the causes of maternal deaths and analyzed substandard care. Recommendations centered on (1) improving maternal death surveillance, (2) installing a maternal mortality review committee to audit every pregnancy-related death, (3) implementing national guidelines and early warning scores, and (4) improving postnatal care strategies.^10^

A RAMoS performed in 2015 retrospectively identified and audited all maternal deaths between 2010 and 2014, and it ultimately led to 4 key recommendations.

To ascertain that the recommendations would be pursued, the study investigators of the 2010–2014 RAMoS sought collaboration with MOH, BOG, PAHO, and midwifery and gynecology/obstetric organizations. Through joint efforts of these stakeholders, the responses that could be implemented were the installation of the committee MaMS and the development of national obstetric guidelines.

## MDSR IMPLEMENTATION PROCESS IN SURINAME BETWEEN 2015 AND 2019

In this section, we describe the perspectives and experiences of the members of committee MaMS, the health care providers, and public health experts (BOG/MOH/PAHO) involved in the MDSR implementation.

### Recommendation Response: Installation of a National Maternal Mortality Review (MaMS) Committee

**FIGURE 2:**
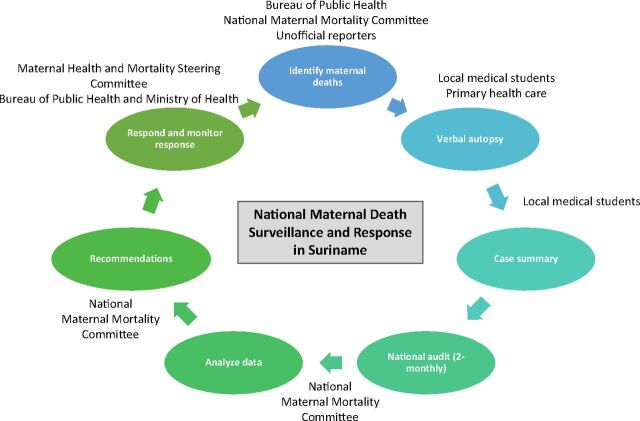
Maternal Death Surveillance and Response in Suriname in 2020 Source: Adapted from the World Health Organization.^7^

The committee MaMS, established in November 2015, gathers (bi)monthly and audits every pregnancy-related death in the nation.^26^ The committee consists of 4 gynecologists/obstetricians, 1 midwife, 1 internal medicine specialist, 1 BOG representative, 2 medical students, and several external consultants.^26^ Most members are consultants from 4 of the 5 major hospitals in Suriname where most births take place; primary health care is not represented. The medical students involved have at least 1 year of experience in obstetrics and discuss every case file with an experienced clinician. Figure 2 depicts the activities currently conducted by the committee MaMS in the MDSR cycle:

1. Active case detection by various sources: (in)formal notification, notification by BOG (C-forms or active surveillance)
2. Sharing of cases (exchange of data) with the BOG and vice versa (not yet performed regularly)
3. Composition of a case summary
4. Collecting additional case information if necessary (e.g., laboratory results, interview with the health care provider)
5. Verbal autopsy with family members if it could contribute more insight into the circumstances of the death
6. Maternal death review/audit, classification using the International Classification of Diseases for Maternal Mortality,^27^ and substandard care analysis according to the 3-delay model ^28^
7. Dissemination of recommendations with relevant institutions and the MOH and BOG (not yet consistently done)

**FIGURE 3:**
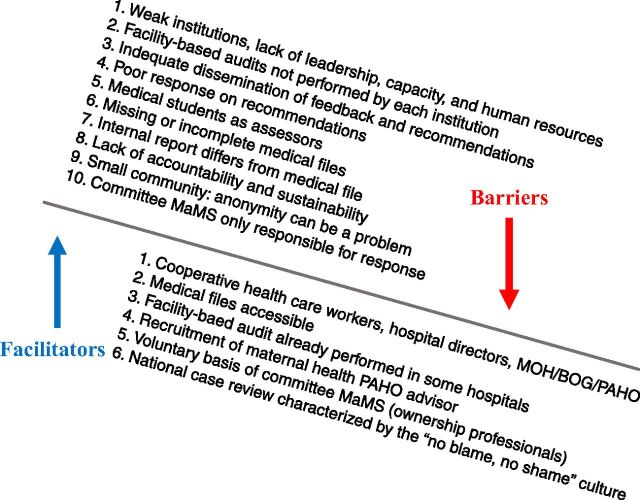
Facilitators and Barriers in Establishing Maternal Death Surveillance and Response in Suriname as Experienced by Committee Maternal Mortality Suriname Members, Health Care Providers, and Public Health Experts Abbreviations: BOG, Bureau of Public Health; MDSR, Maternal Death Surveillance and Response; MaMS, Maternal Mortality Suriname, MOH, Ministry of Health; PAHO, Pan American Health Organization.

Figure 3 summarizes the facilitators and barriers experienced by committee MaMS, health care providers, and public health experts (BOG/MOH/PAHO) in the completion of the MDSR cycle. All audits conducted by committee MaMS guarantee the “no blame, no shame” culture,^5^^,^^29^ and the committee ensures that no litigation of health care workers is initiated.

Unfortunately, despite the efforts of the committee MaMS and PAHO for 5 years now, maternal deaths are still not structurally identified and identification depends on informal notification by health care workers, family, or news sites. Deceased women of reproductive age are not yet completely incorporated in BOG's routine surveillance, facility-based reviews are incidentally performed, and there is no established institution responsible for the general MDSR coordination. The members of the committee MaMS are volunteers. Death certificates do not have a pregnancy box, and notification is not required.^10^ Owing to a lack of trained professionals, medical students are responsible for part of the surveillance, data acquisition, and case presentation at the audit and for summarizing the analysis and recommendations. The level of knowledge of these students may affect the quality of information obtained, confidentiality, and sustainability.

Unfortunately, despite the efforts of the committee MaMS and PAHO, maternal deaths are still not structurally identified.

### Recommendation Response: Obstetric Guideline Development

**FIGURE 4:**
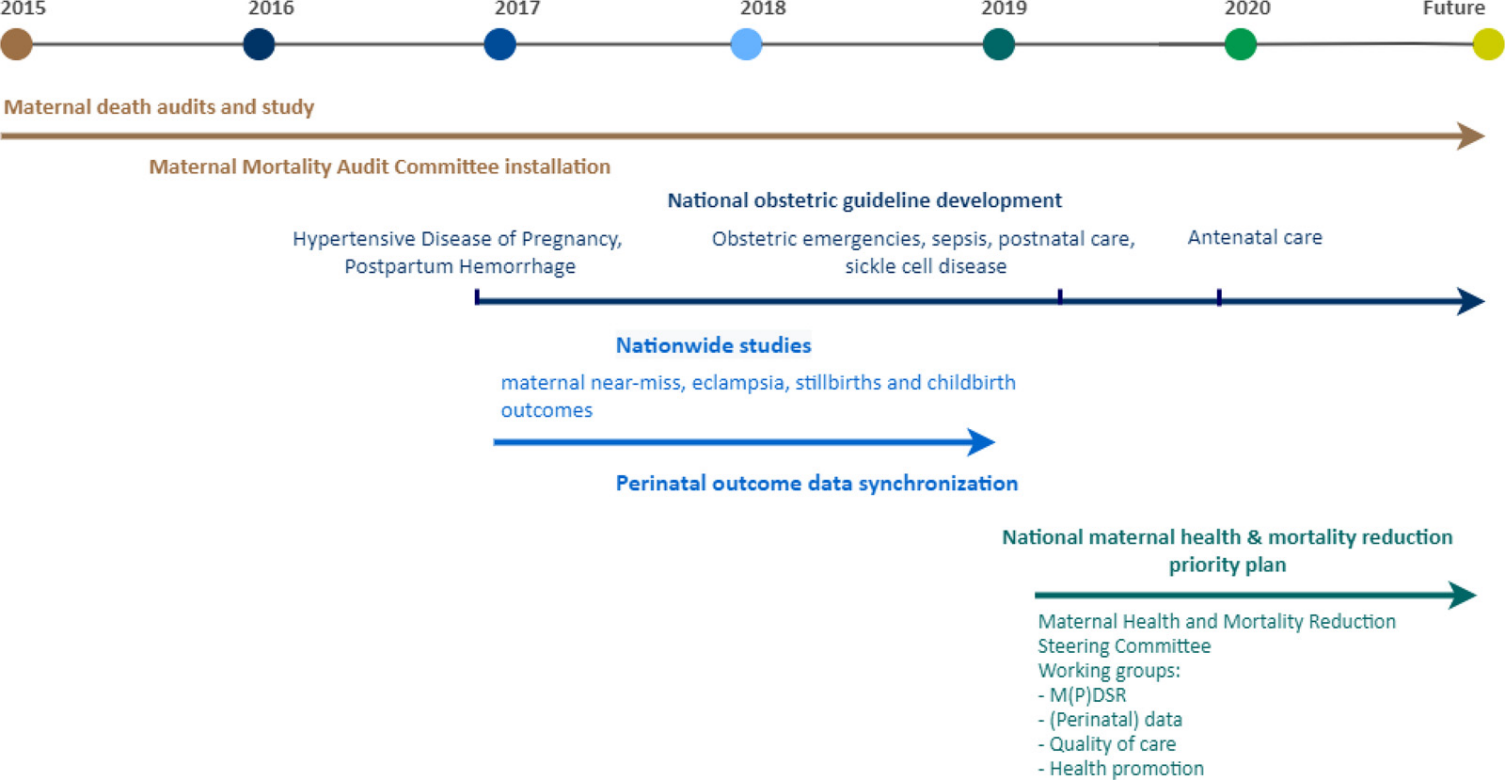
Timeline of Maternal Health Initiatives in Suriname, 2015 to Present Abbreviation: M(P)DSR: maternal (& perinatal) death surveillance and response.

The committee MaMS responded to one of the recommendations on quality-of-care improvement from the 2010–2014 RAMoS in 2016 (Figure 4). This response included an adaptation of international obstetric guidelines to the national context and addressed Suriname's most common maternal health problems, namely postpartum hemorrhage, hypertensive disorders of pregnancy, and obstetric emergency training.^30^ Non-pneumatic anti-shock garments (used in hypovolemic shock in case of severe hemorrhage) were provided by PAHO, followed by training, in 2018 and 2019 to reduce and treat postpartum hemorrhage.^9^ The evaluation of the previous guidelines and the development of guidelines on postnatal and antenatal care, sepsis, sickle cell anemia, emergency obstetrics, and early warning scores followed in April 2019. Facility-based obstetric emergency training was guided by BOG, PAHO, and the recently installed maternal health quality-of-care working group, to enhance guideline implementation and adherence as advised in earlier studies.^31^^,^^32^ In addition to the quality-of-care improvement projects, committee MaMS was involved in conducting nationwide studies on maternal morbidity and near-miss (2017–2019), childbirth outcomes, and stillbirths.^14^^,^^33^

## 2020 AND BEYOND: NEXT STEPS TOWARD FULFILLING THE MDSR CYCLE IN SURINAME

In 2020, MOH formulated different strategies to implement MDSR, based on the recommendations and experiences of the members of committee MaMS, health care providers, and public health experts (BOG/PAHO).

In 2020, MOH formulated different strategies to implement MDSR, based on recommendations and experiences from members of committee MaMS, health care providers, and public health experts.

Similar to Suriname, other countries in the region have not made great progress in reducing maternal deaths.^3^^,^^34^ Subsequently, the PAHO and its Latin American Centre of Perinatology, Women and Reproductive Health called for awareness-raising and accountability.^34^

**FIGURE 5:**
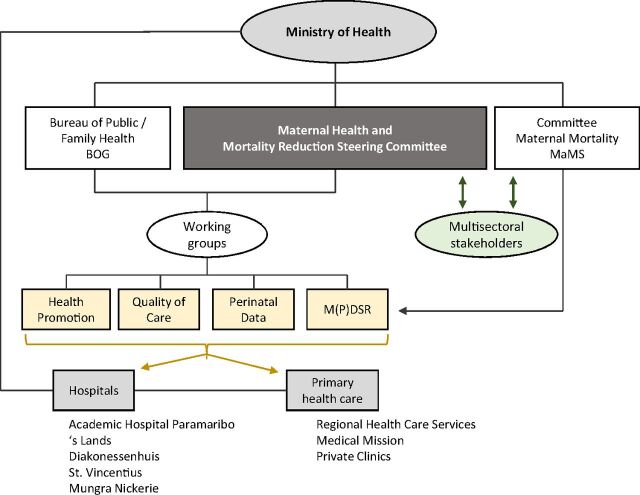
Flowchart of Organization of Maternal Health in Suriname Adapted From the National Maternal Health and Mortality Reduction Priority Plan 2019–2020^36^

MOH/BOG and PAHO presented an advocacy paper and priority plan in April 2020 to call for a multisectoral effort to reduce maternal deaths.^35^^,^^36^ They also created a national steering committee for maternal health and mortality reduction to reinforce the coordination of the maternal health program in Suriname.^36^ This steering committee was formally installed by MOH in February 2020, and it currently guides, advises, and closely monitors planned interventions of the working groups and reinforces accountability and multisectoral coordination. Although the committee MaMS (responsible for review and recommendations) previously also carried out its own recommendations, MOH and BOG agreed that this steering committee, a direct working arm of MOH, should be responsible for the coordination and monitoring of response. The steering group oversees 4 working groups to ensure more commitment (Figure 5).^36^ Through these working groups, responsibilities can be specifically delineated and roles defined. In particular, because of the lack of leadership, it is important to define exactly who is responsible and what the responsibilities are. The 4 working groups include the following:

1. The MDSR working group is responsible for improvements in *surveillance* and maternal death audit, dissemination of *recommendations*, and delineation of roles for *response* by specifying specific tasks and responsibilities.
2. The quality-of-care working group is responsible for the development and monitoring of national standards of care. It also updates and validates national guidelines and supports facility-based and national training.
3. The perinatal (data) working group is responsible for introducing, collecting, synchronizing, and analyzing data on perinatal health in Suriname.
4. The health promotion working group is responsible for the development of a health promotion plan. It implements recommendations following maternal death reviews and supports maternal health education, family planning, and contraception in the communities.

MOH has also identified multisectoral focal points in non-health ministries and institutions and currently prepares the national Maternal and Neonatal Health Strategy (2021–2025) and Operational Plan (2021–2023). Unfortunately, the COVID-19 pandemic has caused a delay in action and operation of the working groups.

### Strategies to Institutionalize MDSR in Suriname

To guarantee sustainable surveillance and to improve *identification and notification* of maternal deaths, MDSR focal points (midwives/doctors) are designated in each institution (the 5 hospitals, Medical Mission, and Regional Health Services).^36^ The MDSR focal point in a hospital is responsible for active case detection by monthly medical file investigation of deceased women of reproductive age. The primary care MDSR focal point assesses community deaths. MOH issued instructions on the procedure for early reporting and active case detection to health facilities and burial agencies. In addition, PAHO and the Latin American Centre of Perinatology, Women and Reproductive Health organized training in active case detection, verbal autopsy, and review to improve MDSR.

Following the identification of a possible maternal death, BOG must be notified via a hotline number, and the case must be entered in an anonymized password-protected online database. If no maternal deaths occur, that information must also be reported. The focal point is responsible for the coordination of a more structured facility-based review and reports to BOG and committee MaMS. Although some facilities conduct maternal death reviews within 72 hours, conclusions and lessons learned can differ from the national review. Anonymity and the “no blame, no shame” culture cannot be assured in the facilities. Additional barriers include the lack of leadership to review these deaths and no obligation or request for a report for MOH.

Therefore, it is crucial to perform an external case assessment by specialized trained nurses or medical doctors from BOG with the assistance of committee MaMS. The monthly audits to determine underlying causes and classification on the national level by committee MaMS should continue. Committee MaMS formulates the recommendations and disseminates them to the relevant institutions and MOH/BOG. Although the committee MaMS instigated the previously mentioned responses, they do not have the authority to act upon recommendations, for example, by adding a pregnancy check box on the death certificates, enacting a national policy to ensure notification of maternal deaths, and mandating postmortem investigation of all unexplained maternal deaths. Therefore, we believe that the response to the recommendations from the review cannot only be the responsibility of committee MaMS. Strong government commitment and leadership of professionals are essential to ensure response on the recommendations, evaluation, and monitoring and to judge the impact on maternal death reduction. Table 1 provides an overview of the implementation status of the abovementioned strategies in 2020.

**TABLE 1.** Summary of the Implementation Status of Maternal Death Surveillance and Response in 2020

Already Established	To Be Established
Installation of a national review committee (MaMS) and a maternal health mortality reduction steering committee Coordination framework and terms of references Institutional MDSR focal points designated and trained in surveillance and active case detection Quality-of-care working group operational	National policy for notification of maternal deaths Official installation of committee MaMS and the 4 working groups for maternal health, reinforce the health promotion and perinatal data working group^a^ Facility-based audits of every case, organized by MDSR focal points Verbal autopsies by MDSR focal points^b^ Specialized assessors for facility audit preparation (nurses, doctors) and external audits (BOG) Timely dissemination of recommendations Monitoring and evaluation

Abbreviations: BOG, Bureau of Public Health; MaMS, Maternal Mortality Suriname; MDSR, Maternal Death Surveillance and Response.

a Perinatal data working group was installed, but only sporadically active.

b Only incidentally performed until now.

### Recommendations to Strengthen MDSR in Suriname

In Table 2, we summarize the recommendations from committee MaMS members, health care providers, and public health experts following the lessons learned since the implementation of MDSR in Suriname in 2015. Critical steps in fulfilling the complete MDSR cycle in Suriname (action and response) are delineating roles and responsibilities for action, establishing accountability mechanisms for results, and influencing stakeholders in a position to act. The fulfillment of this cycle is hindered by a lack of financial and human resources, leadership, and legislation and by inadequate government enabling policies.

**TABLE 2.** Summary of Recommendations From Committee Maternal Mortality Suriname Members, Health Care Providers, and Public Health Experts to Strengthen Maternal Death Surveillance and Response in Suriname

Legislation
Ensure no disciplinary/litigation measures Notification of maternal death within 24 hours^a^ Include pregnancy checkbox on the death certificate^b^ Timely completion of death certificate Autopsy for maternal deaths of unknown cause
**Finances and human resources**
Empower BOG by capacity strengthening Support committee MaMS (administrative personnel, logistics) Capacity building of the institutional MDSR focal points Include MDSR in preservice training curricula Involve health care workers and create awareness (bottom-up approach) Involve and educate the community Funding
**Enabling policies**
Ensure structural facility-based review Install special secretariat for MDSR at BOG/MOH Enable communication and dissemination of findings and recommendations

Abbreviations: BOG, Bureau of Public Health; MaMS, Maternal Mortality Suriname; MDSR, Maternal Death Surveillance and Response; MOH, Ministry of Public Health.

a Institutions and funeral agencies were recently requested to report maternal deaths within 24 hours.

b As a temporary solution, a pregnancy checkbox slip is attached to the “C form.”

### MDSR in the Future: Adding Perinatal Deaths to the Cycle

**FIGURE 6:**
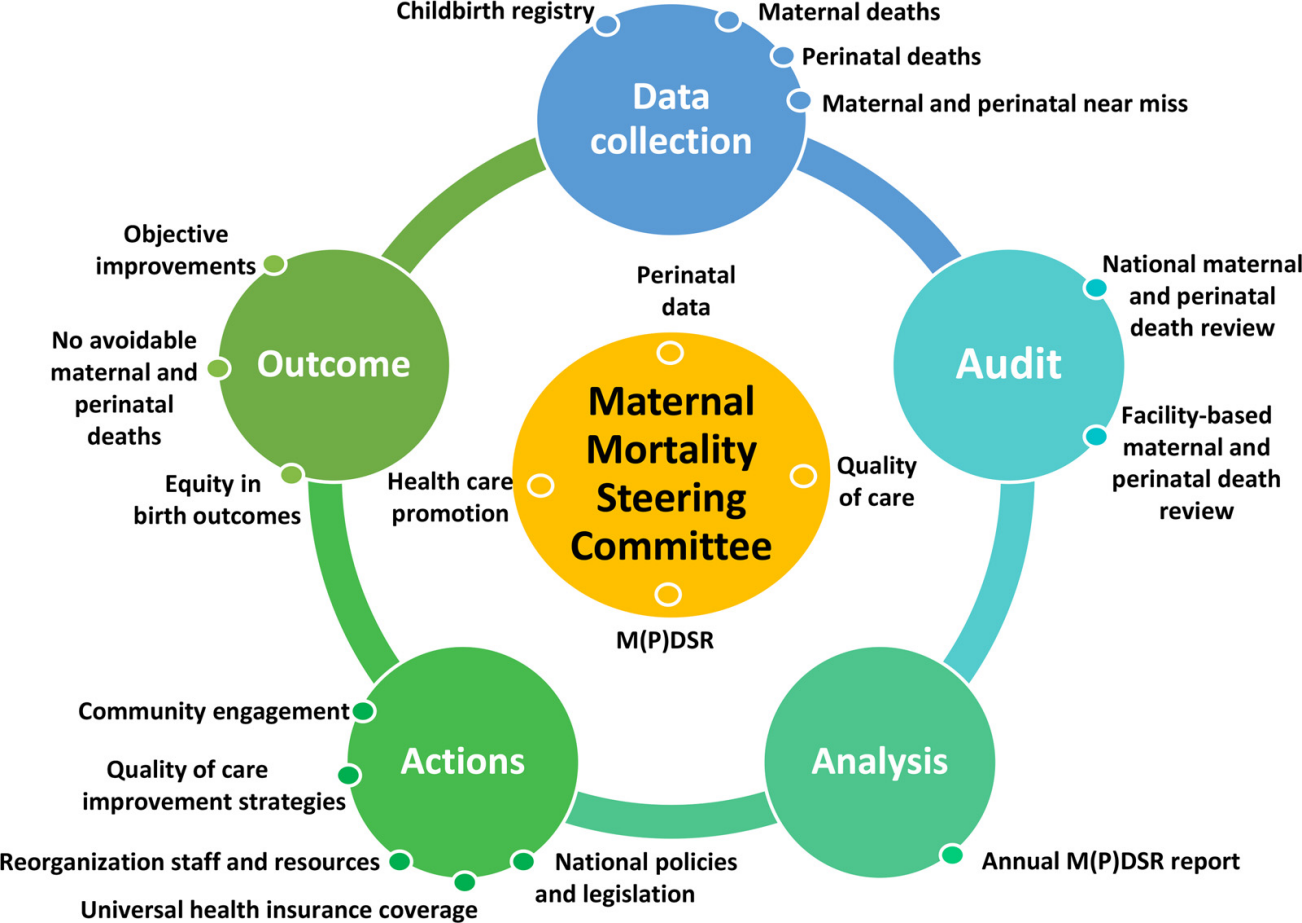
The Ideal Paradigm of the Maternal and Perinatal Death Surveillance and Response Cycle for Suriname Abbreviation: MPDSR, maternal and perinatal death surveillance and response.

The following step after the institutionalization of MDSR implementation will be the inclusion of perinatal deaths to the cycle, the Maternal and *Perinatal* Death Surveillance and Response (M*P*DSR) (Figure 6). Maternal conditions often influence perinatal outcomes.^37^^,^^38^ Additionally, gathering perinatal data and performing perinatal mortality audits in the future will extend the MDSR cycle and link maternal and perinatal care. Further, maternal morbidity and near-miss data gathering and audits constitute another essential step. We realize, however, that perinatal deaths are more numerous and the implementation of perinatal death surveillance and review will therefore be more challenging. The plans are to start with a pilot to systematically audit perinatal deaths in 2021–2022.

## CONCLUSIONS

For decades, several attempts by MOH alone were insufficient to institutionalize maternal death audits. Structural national maternal death review in Suriname was introduced after a timely and complicated process. Stakeholders' involvement, ownership, and leadership were essential to step up in the MDSR cycle from insufficient surveillance to structural audits in 2015. These first steps created a base that the institutions in charge can build on to ensure sustainability. Therefore, a strongly committed government enabling clear policies and laws to improve MDSR is crucial. In summary, the key elements for successful MDSR implementation are commitment, “no blame, no shame” culture, collaboration, coordination, and communication (the 5 Cs).

## Notes

**Peer Reviewed**
